# Use of Heart Rate Variability Biofeedback to Reduce the Psychological Burden of Frontline Healthcare Professionals Against COVID-19

**DOI:** 10.3389/fpsyg.2020.572191

**Published:** 2020-10-30

**Authors:** Juan-Pablo Aristizabal, Raphael Navegantes, Eline Melo, Antonio Pereira

**Affiliations:** ^1^Graduate Program in Neuroscience and Behavior, Federal University of Pará, Belém, Brazil; ^2^Graduate Program in Electrical Engineering, Federal University of Pará, Belém, Brazil; ^3^Graduate Program in Cell Biology and Neuroscience, Federal University of Pará, Belém, Brazil

**Keywords:** COVID-19, biofeeback, heart-rate variability (HRV), healthcare personnel, mental health

Fear of getting infected and infecting other people, feeling responsible for the physical and mental well-being of their patients, working in a novel and unpredictable context subject to work overload and shortage of personal protective equipment are just a few of the difficult situations that frontline healthcare professionals are facing in the ongoing fight against COVID-19 (Figure 1A) (Liu et al., 2020). When this experience is superimposed on the typical baseline stressors of the profession such as low morale and low wages, it can contribute to increasing the burden of mental health problems experienced by healthcare professionals during the pandemic and will probably persist even after the COVID-19 crisis has passed. According to Lai et al. (2020), of 1,257 health workers involved with the diagnosis and treatment of COVID-19 patients who were surveyed in China, a considerable proportion experienced symptoms of anxiety (44%), depression (50%), insomnia (34%), and general distress (71%). A similar study carried out in Italy points to the same results: out of 1,379 health professionals surveyed, a high proportion presented symptoms associated with posttraumatic stress disorder (49%), major depressive disorder (25%), anxiety (20%), insomnia (8%), and perceived stress (22%) (Rossi et al., 2020). Posttraumatic stress disorder (PTSD), in particular, though commonly linked with war veterans, is expected to have a surge of occurrences in frontline health professionals after the pandemic (Dutheil et al., 2020). This adds to the realization that both during and after a pandemic, the number of people affected in their mental health tend to be greater than the number of people affected by the infection itself (Reardon, 2015). HIV, Ebola, Zika, H1N1, SARS, and MERS are just a few recent examples of pandemic diseases with such characteristics (Kisely et al., 2020; Ornell et al., 2020).

**Figure 1:**
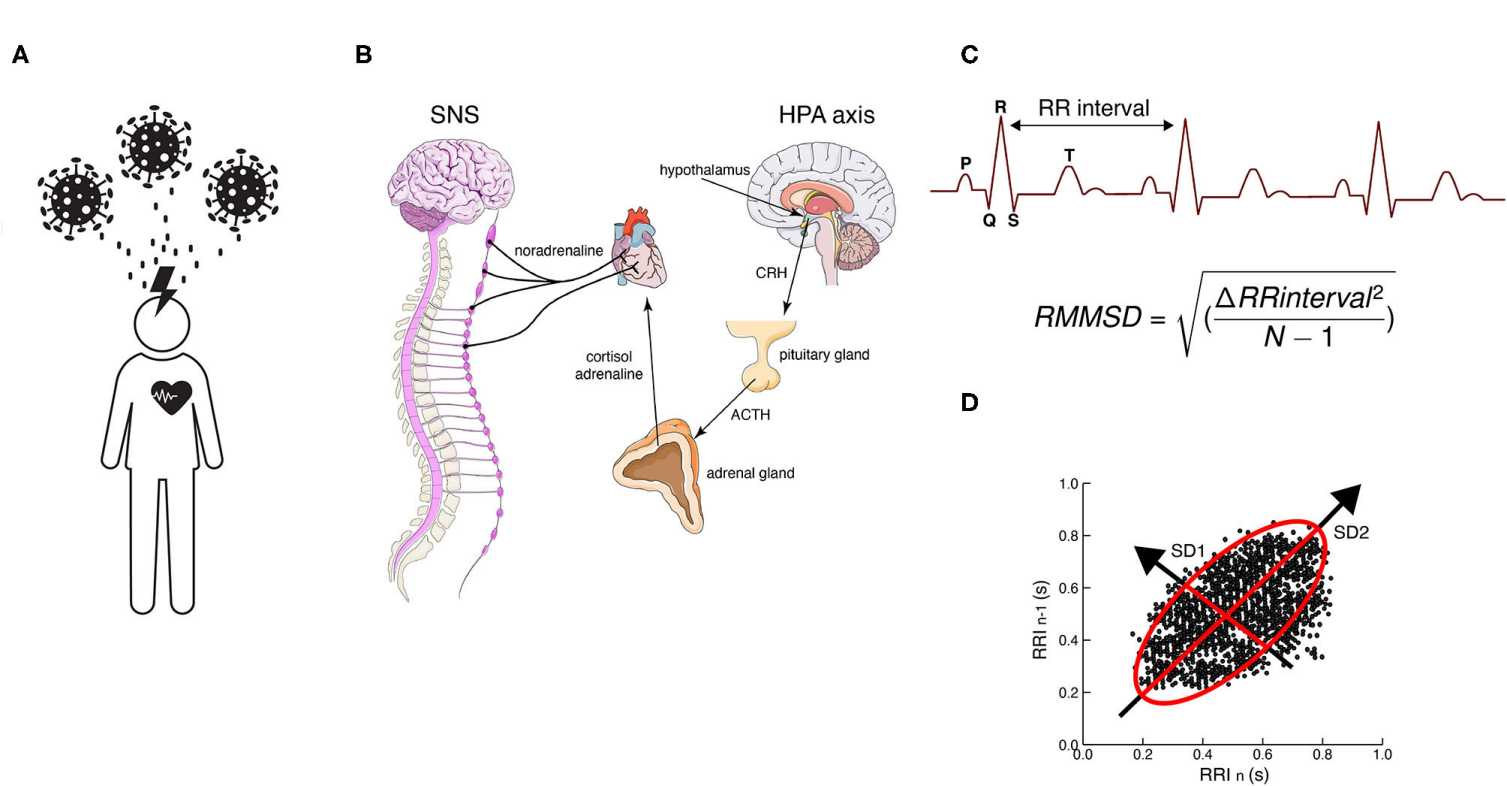
**(A)** Work-related chronic stress of professional healthcare workers due to COVID-19. **(B)** Hypothalamus–pituitary–adrenal (HPA) axis activation due to chronic stress conditions caused by COVID-19. Time-domain tools used to assess HVR **(C)** RMSSD (root mean square of successive differences) and **(D)** Poincaré plots, a geometrical and nonlinear tool to assess the dynamics of HRV. Both evaluate R-R interval variability. CRH, corticotropin-releasing hormone; ACTH, adrenocorticotropic hormone; SNS, sympathetic nervous system.

An acute stressful situation causes the immediate activation of the sympathetic nervous system (SNS) and the hypothalamus–pituitary–adrenal axis (HPA) and kicks off the release of catecholamines (adrenaline and noradrenaline) and cortisol in the bloodstream that prepares the body for action, enabling physiological and behavioral fight or flight responses geared for the organism's survival (Godoy et al., 2018) (Figure 1B). These responses include heart rate acceleration, increased myocardial contraction force, arterial vasodilation in skeletal muscles, arterial vasoconstriction in the digestive system, and relaxation of smooth muscles in the pupils and bronchi, among others (Mendoza and Foundas, 2007). The body stays on high alert as long as cortisol and adrenaline levels remain high. After a while, the parasympathetic nervous system (PNS) brakes those responses through the vagus nerve and promotes the “rest and digest” phase that restores the body after the danger has subsided.

Healthcare professionals facing high-stress situations are likely to present harmful physiological adaptations associated with overactivation of the SNS. Though the body can quickly react to stressful situations through the HPA axis, many disease states are characterized by chronically elevated sympathetic nerve activity (SNA) (Fisher et al., 2009). The body's inability to return to basal homeostatic levels of both catecholamines and cortisol in the bloodstream caused by chronic stressors can have devastating wear and tear effects on the cardiovascular, digestive, immune, and nervous systems (Dünser and Hasibeder, 2009). In the current pandemic situation, which will probably continue until an effective vaccine arrives, it is important to ask how this crisis is affecting the mental health of healthcare professionals and how we can help them to avoid future chronic health complications due to chronic overactivation of the fight or flight response.

In a healthy person in a resting state, the heartbeat frequency is not regular but changes constantly due to sympathetic/parasympathetic regulation. Heart rate patterns are normally determined by the tonic functional outflow from the vagus nerve to the heart (i.e., cardiac vagal tone) (Porges, 1995). The heart rate variability (HRV), or the time variation between consecutive heartbeats, is an emerging property of autonomic regulatory systems operating at different time scales and helping the body adapt to different environmental and psychological challenges. The normal range of HRV depends on the interaction between sympathetic and parasympathetic inputs to the heart (Lombardi and Stein, 2011). While increased HRV is usually associated with good health conditions, lowered HRV is an indicator of risk related to various pathologies (Lopes and Palmer, 1976).

The neurovisceral integration (NVI) model (Thayer and Lane, 2000; Thayer et al., 2009) proposes that adaptive behavior depends on the integration of neural networks spanning both the central (CNS) and autonomic nervous systems (ANS) tasked with regulating cardiovascular function. Thus, there is a bidirectional communication pathway between the ANS and the CNS providing a dynamic regulation mechanism in which brain structures affect the functioning of visceral organs, and these, in turn, send afferent sensory information to the brain affecting its function (Hess, 1949).

Since the 1980s, biofeedback-based intervention tools have been developed, which aim to train people in the voluntary control of physiological parameters through audiovisual feedback mechanisms. There are several types of biofeedback approaches based on different physiological signals such as electromyography, peripheral body temperature, and heart rate variability (HRV-B) (Lehrer and Gevirtz, 2014). HRV-B aims to stimulate efferent vagal activity and induce respiratory sinus arrhythmia (RSA) through repeated exercises of diaphragmatic respiration control (Porges and Kolacz, 2018), resulting in increased HRV (Shaffer and Ginsberg, 2017). RSA is the normal variation in heart rate that accompanies breathing: inhalation temporarily suppresses vagal activity, decreasing the time between heartbeats and increasing the heart rate, while exhalation produces the opposite effect. The practice of HRV-B induces the person to breathe in a low frequency (~10 breaths per minute), lengthening the exhalation period to increase the amplitude of the RSA and the HRV (Lehrer and Gevirtz, 2014). The final goal of this procedure is to increase the flexibility and recovery capacity of the cardiovascular system facing stressful situations, allowing the individual to return to homeostatic equilibrium states (Gevirtz, 2013). A recent study showed that even a single session of HRV- B was able to increase HRV (Lin et al., 2020).

Two proposed mechanisms underlie HRV-B training. The first is the induction of the baroreflex—a rapid negative feedback loop in which elevated blood pressure due to inspiration decreases heart rate and blood pressure (Lombardi and Stein, 2011). The second is based on the idea that oscillatory rhythms associated with the respiratory drive influence oscillatory patterns in the vagal and sympathetic outflows (Lopes and Palmer, 1976). Due to the relationship between heart rate and breathing, HRV-B can also improve efficiency in respiratory gas exchange. Due to the fact that HRV-B can improve blood pressure control through baroreflex and vagal stimulation while inducing feelings of relaxation and well-being (Lehrer et al., 2020), it has become a very popular method of psychological intervention in recent years (Lehrer and Gevirtz, 2014). For instance, it has been proven to alleviate anxiety symptoms in students (Lee et al., 2015), posttraumatic stress in war veterans (Schuman and Killian, 2019), and depressive symptoms on persons with major depressive disorder (Caldwell and Steffen, 2018), and also improve cognitive, artistic, and sports performance (Lehrer et al., 2020). The HRV is obtained from electrocardiogram (ECG) measurements and the different parameters of HRV are obtained in both the time and frequency domain. Usually, the easiest and fastest way to represent vagally mediated changes in HRV is with time-domain variables, such as the root mean square of successive differences between normal heartbeats (rMSSD) (Shaffer and Ginsberg, 2017) (Figures 1C,D). The rMSSD is the main feature used in mobile HRV applications because it is easy to acquire and compute with short time measures (Penttila et al., 2001).

During an HRV-B training session, the person may be instructed to sit or lie supine in a relaxed position and to maintain diaphragmatic respiration rates between 6 and 10 breaths per minute, while being guided by real-time feedback display of their heart rate and respiration rate. This feedback can be gamefied and be adjusted according to the evaluated parameters and represent the success or failure of the training. Eventually, the person should become aware of the control they can exercise over autonomous processes such as HRV (Caldwell and Steffen, 2018).

This intervention is becoming increasingly attractive as therapeutic support probably due to the latest developments in portable devices, which have increased its accessibility and practical utility in different contexts. While some mobile applications may require the purchase of specialized external sensors (Goessl et al., 2017), others rely on smartwatches (Hernando et al., 2018) and even cell phone cameras (Peng et al., 2015; Bánhalmi et al., 2018). In any case, these electronic consumer devices are easy to use and allow the design of personal training programs adjusted to age, sex, height, weight, and physical aptness. They are implemented with different types of feedback in the form of games, videos, and sounds and allow the export of data for visualization and traceability of training history (Peake et al., 2018).

Though the negative mental health effects of COVID-19 are not restricted to healthcare professionals, the fight against the pandemic depends on their being capable to perform their jobs optimally without compromising their health. Supporting the mental health of these individuals is a critical part of the public health response to COVID-19. Most healthcare organizations traditionally put their resources toward supporting staff only once they have developed a mental health pathology. However, beyond treating the disease, it is important to promote prevention campaigns focused on mitigating the psychological impact of the pandemic (Walton et al., 2020). Thus, it is important to mobilize all available resources to help healthcare workers to fulfill their professional obligations and keep being available for the prolonged fight against COVID-19 and many other threats facing humankind in the future. Given the challenges of social distancing, easily available technological tools are an important adjunct to traditional psychological therapies, and HRV-B training is an accessible way to help reduce the mental toll imposed by COVID-19 on frontline professionals.

Even if you do not have access to HRV-B, ideally assisted by a trained professional, you still can perform diaphragmatic breathing exercises, which have also been shown to have positive effects in the reduction of feelings of stress and anxiety through modulation of HRV (Ma et al., 2017).

Diaphragmatic or abdominal paced breathing is the conscious use of your diaphragm to breathe at a rate of 10 times per minute while making sure to exhale longer than you inhale (Szulczewski, 2019).

For this exercise you must:

1- Find a comfortable and quiet place.
2- Sit in a comfortable chair or lie on your back with a pillow under your head.
3- Place one hand on your chest and the other on your abdomen.
4- Close your lips and slowly inhale through the nose, counting to 4 in your head (during inhalation, the abdomen must raise the hand and your chest must remain still).
5- Expel the air slowly through your mouth, counting to 6 (as you expel the air, you should feel your abdomen sink).
6- Practice this breathing technique for 5 to 10 min and try to perform it during your breaks 3 to 4 times a day.

## Author Contributions

All authors listed have made a substantial, direct and intellectual contribution to the work, and approved it for publication.

## Conflict of Interest

The authors declare that the research was conducted in the absence of any commercial or financial relationships that could be construed as a potential conflict of interest.
